# Preferred crystallographic orientation of cellulose in plant primary cell walls

**DOI:** 10.1038/s41467-020-18449-x

**Published:** 2020-09-18

**Authors:** Dan Ye, Sintu Rongpipi, Sarah N. Kiemle, William J. Barnes, Arielle M. Chaves, Chenhui Zhu, Victoria A. Norman, Alexander Liebman-Peláez, Alexander Hexemer, Michael F. Toney, Alison W. Roberts, Charles T. Anderson, Daniel J. Cosgrove, Esther W. Gomez, Enrique D. Gomez

**Affiliations:** 1 Department of Chemical Engineering, The Pennsylvania State University, University Park, PA 16802 USA; 2 Department of Biology, The Pennsylvania State University, University Park, PA 16802 USA; 3 Department of Biological Sciences, The University of Rhode Island, Kingston, RI 02881 USA; 4 Advanced Light Source, Lawrence Berkeley National Laboratory, 1 Cyclotron Road, Berkeley, CA 94720 USA; 5 Stanford Synchrotron Radiation Lightsource, SLAC National Accelerator Laboratory, Menlo Park, CA 94025 USA; 6 Department of Biomedical Engineering, The Pennsylvania State University, University Park, PA 16802 USA; 7 Department of Materials Science and Engineering and Materials Research Institute, The Pennsylvania State University, University Park, PA 16802 USA; 8 Present Address: 123 Clapp Laboratory, Mount Holyoke College, 50 College Street, South Hadley, MA 01075 USA

**Keywords:** Plant sciences, Cell wall, Materials science, Biomaterials

## Abstract

Cellulose, the most abundant biopolymer on earth, is a versatile, energy rich material found in the cell walls of plants, bacteria, algae, and tunicates. It is well established that cellulose is crystalline, although the orientational order of cellulose crystallites normal to the plane of the cell wall has not been characterized. A preferred orientational alignment of cellulose crystals could be an important determinant of the mechanical properties of the cell wall and of cellulose-cellulose and cellulose-matrix interactions. Here, the crystalline structures of cellulose in primary cell walls of onion (*Allium cepa*), the model eudicot Arabidopsis (*Arabidopsis thaliana*), and moss (*Physcomitrella patens*) were examined through grazing incidence wide angle X-ray scattering (GIWAXS). We find that GIWAXS can decouple diffraction from cellulose and epicuticular wax crystals in cell walls. Pole figures constructed from a combination of GIWAXS and X-ray rocking scans reveal that cellulose crystals have a preferred crystallographic orientation with the (200) and (110)/($1\bar 10$) planes preferentially stacked parallel to the cell wall. This orientational ordering of cellulose crystals, termed texturing in materials science, represents a previously unreported measure of cellulose organization and contradicts the predominant hypothesis of twisting of microfibrils in plant primary cell walls.

## Abstract (web summary)

Cellulose is synthesized as microfibrils of β-1,4-linked glucan chains arranged in a crystalline lattice. Here Ye *et al*. use grazing incidence wide angle X-ray scattering to show that cellulose crystals are preferentially orientated parallel to the plant cell wall, rather than as twisting microfibrils as previously hypothesized.

## Introduction

Cell walls of growing plants are dynamic, heterogeneous hydrated networks comprised of cellulose, hemicellulose, pectin, and structural proteins. The spatial organization of these components in the cell wall is an important determinant of anisotropic growth and mechanical properties in plants^1–3^. Cellulose is synthesized by enzyme complexes in the plasma membrane as elementary microfibrils^4^, here termed microfibrils, consisting of 18 extended, β-1,4-linked glucan chains^5,6^. X-ray diffraction (XRD) reveals that the glucan chains are arranged in a crystalline lattice^7–9^. The crystalline structure of cellulose impacts biological processes controlling plant growth, efficiency of conversion of cellulosic biomass into renewable energy, and physical properties of cellulose-derived materials.

In cell walls, cellulose ordering exists over a wide range of length scales. At the smallest scale, cellulose order is defined by how glucan chains crystallize through hydrogen bonding and van der Waals interactions^10^. Cellulose crystals adopt a microfibril habit, whose reported diameters are often between 3 and 4 nm^11^. Many primary cell walls have a crossed polylamellate structure in which microfibrils are oriented in a common direction within a lamella, but orientation varies between adjacent lamellae^12,13^. At larger scales, the polylamellate structure may exhibit a net microfibril alignment at an angle relative to the elongation axis of the cell^14–16^. Several studies have reported these forms of cellulose ordering in plant cell walls of onion, pineapple, cabbage, wheat straw, and inflorescence stems of *Arabidopsis thaliana* through various techniques, such as solid-state ^13^C nuclear magnetic resonance (NMR) spectroscopy, atomic force microscopy (AFM), X-ray scattering, and sum frequency generation (SFG) spectroscopy^10,14–16^. The distribution and alignment of cellulose microfibrils within the cell wall contributes to mechanical anisotropy, which enables directional cell expansion^17^.

Another possible type of cellulose ordering, largely unexamined, is the orientation of cellulose crystallites relative to the plane of the cell wall and thus relative to other crystallites as well. Crystal planes orienting along a specific direction over a long range such that they exhibit a preferred crystallographic direction is called “texturing.” Orientational order of cellulose crystallites with respect to the plane of the cell wall has rarely been examined in primary cell walls. Lack of this orientational order may suggest twisting of cellulose microfibrils (such twist has been previously predicted in primary cell walls^12,18^) or a random orientation of cellulose crystals during cell wall deposition or wall assembly. As such, identifying a preferred orientational order with respect to the plane of the cell wall would imply an aspect of cell wall assembly not previously considered and may have consequences on how we describe the link between microstructure and mechanical properties of primary cell walls.

Grazing incidence wide-angle X-ray scattering (GIWAXS) is a structural characterization tool that is commonly used to study the preferred crystal orientation in polymer and nanoparticle thin films^19–23^. In this technique, the X-ray beam is incident to the sample at a shallow angle. Working in a grazing incidence geometry near the critical angle for total external reflection, often between 0.1° and 0.2° for carbonaceous materials, enhances the external electric field at the sample, increases the X-ray path length on the sample, and thereby increases the scattering signal^24^. Furthermore, using a two-dimensional (2D) detector reveals the orientation of crystallites, such as the texture of crystals in orientations that are in plane, out of plane, or at some intermediate angle with respect to the sample surface. GIWAXS of plant cell walls can provide information about chain packing along the direction of and normal to the plane of the cell wall.

We examined the orientation of cellulose crystals with respect to the plane of primary cell walls of onion epidermis, Arabidopsis hypocotyls, and moss leaves (phyllids) using GIWAXS. By capturing diffraction in the direction parallel and perpendicular to the cell wall plane, GIWAXS decouples diffraction from cellulose and epicuticular wax crystals. Clear evidence of a preferred crystal orientation for cellulose is observed, where the (200) and (110)/($1\bar 10$) planes are preferentially stacked parallel to the cell wall. The net isotropy of in-plane microfibril alignment allows us to quantify the degree of preferred crystal orientation of cellulose in cell walls through *χ*-pole figures constructed from a combination of GIWAXS and X-ray rocking scans. The strong orientational ordering of cellulose crystals detected here is inconsistent with previously predicted twisting of cellulose crystals in plant primary cell walls^12,18,25,26^. We expect that crystal texture, a measure of cellulose organization, will aid in linking nanoscale and microscale cellulose structure to the mesoscale organization of cellulose microfibrils and to macroscale mechanical properties of plant cell walls.

## Results

### Transmission Wide-Angle X-ray Scattering (WAXS) of onion primary cell walls

Transmission WAXS has been used to reveal alignment of cellulose microfibrils in well-ordered cell walls, such as spruce wood^7^ and celery collenchyma^12^. We used WAXS measurements of unextracted and chloroform-treated onion epidermis, where the incident X-ray beam is perpendicular to the cell wall plane, to probe the possibility of alignment of crystal planes of cellulose along the plane of the cell wall due to a net alignment of microfibrils. The scattering pattern obtained from the unextracted onion epidermal cell wall is largely dominated by bright sharp rings at scattering vectors *q* of about 1.52 and 1.70 Å^−1^ (*q* = 2*π*/*d*, *d* is the lattice spacing), which are not seen in the scattering from a chloroform-treated onion epidermal cell wall (Supplementary Fig. 1).

The onion epidermis consists of a cell wall layer and a cuticle layer. The cuticle contains heterogeneous polymers including cutin, cutan, and epicuticular wax crystals^27,28^. Previous electron and XRD studies have reported lattice spacings of epicuticular wax as 4.13 and 3.73 Å^29^, which correspond well to *d*-spacings of 4.13 Å and 3.70 Å obtained from transmission WAXS data of unextracted onion cell wall (Supplementary Fig. 1a). Chloroform treatment of cell wall samples, which is known to remove wax^27,28,30,31^, results in the disappearance of the bright rings in WAXS data (Supplementary Fig. 1b), likely indicating the loss of wax crystals. The broad scattering feature at *q* between about 1.0 and 1.6 Å^−1^ is consistent with Bragg reflections of cellulose Iβ in primary cell walls^9,12^. Scattering intensities shown in Supplementary Fig. 1b are isotropic along the ring, suggesting an average random orientation of cellulose crystals within the cell wall plane. This is likely due to the polylamellate cell wall, where cellulose microfibrils have a preferred direction along each lamella, but the orientation of cellulose microfibrils varies between different lamellae^13^.

### GIWAXS decouples scattering from cellulose and epicuticular wax crystals

**Fig. 1:**
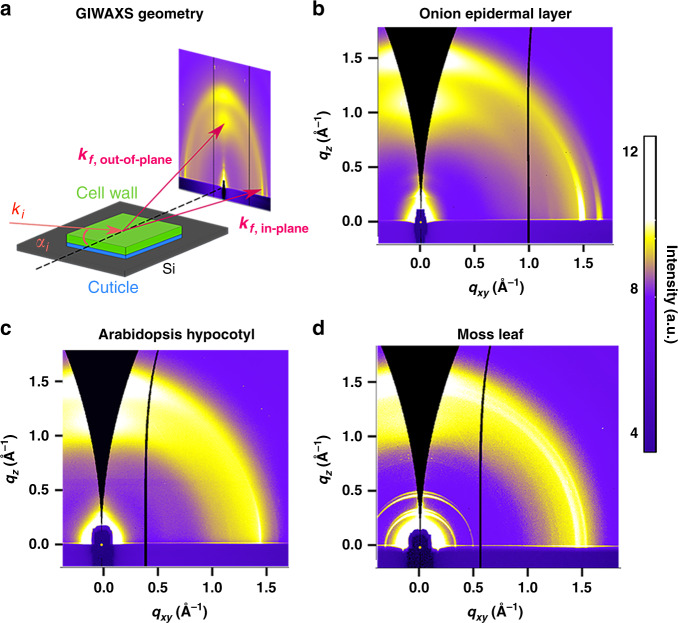
GIWAXS 2D data reveal anisotropic scattering from plant primary cell walls. **a** GIWAXS geometry for examination of the onion epidermal cell wall (*α*_*i*_: angle of incidence; *k*_*i*_: incident wavevector; *k*_*f*,in-plane_: in-plane scattered wavevector; *k*_*f*,out-of-plane_: out-of-plane scattered wavevector). GIWAXS data from **b** unextracted onion epidermis, **c** unextracted Arabidopsis hypocotyls, and **d** unextracted moss.

GIWAXS measurements can reveal diffraction along and perpendicular to the cell wall plane, as shown in Fig. 1a. Plant tissues were mounted on silicon substrates with the cell wall side face up and the cuticle side down in the case of onion. Arabidopsis hypocotyls and moss leaves were also mounted flat on a silicon wafer as whole tissues, as described in the “Methods” section. Scattering is enhanced near the critical angle for total external reflection, which we can estimate using the density. The tissues are mostly made up of primary cell wall, itself composed of cellulose, pectin, and hemicellulose (i.e., mostly composed of hexoses and pentoses), with similar densities (cellulose^32^ 1.5 g/cm^3^, pectin^33^ 1.5 g/cm^3^, and hemicellulose^34^ 1.52 g/cm^3^). The density of our samples was calculated by assuming that primary cell wall is composed of 40% cellulose, 30% pectin, and 30% hemicellulose^35^. This gives a cell wall density of 1.5 g/cm^3^, which is consistent with a previous report of the density of dried cell wall^36^. Thus, at X-ray energies of 10 and 12 keV, critical angles are 0.148° and 0.116°, respectively. Despite the roughness of cell walls, e.g., approximately 10 nm root mean square from AFM images of onion cell wall previously reported^37^, GIWAXS intensities peak at these incidence angles (Supplementary Fig. 2). For our experiments, we use incident angles of 0.15° for 10 keV X-rays and 0.12° for 12 keV, leading to penetration depths of 1.32 and 1.67 μm into the cell wall, respectively. Given that the thicknesses of the dried cell walls examined are about 3.4 μm for onion^38^, 2.5 μm for Arabidopsis hypocotyls, and 10 μm for moss leaves^39^ as measured by profilometry and electron microscopy, these penetration depths probe a significant portion of the cell wall.

GIWAXS 2D images of the onion epidermal cell wall, Arabidopsis hypocotyls, and moss phyllids reveal anisotropic scattering as shown in Fig. 1b–d, respectively. Because of the grazing incidence scattering geometry, the detector cannot capture the entire Ewald sphere, and GIWAXS images need to be geometrically corrected^24^. This leads to a partial lack of data in the out-of-plane direction as denoted by dark triangular regions in Fig. 1b–d. As discussed below, data were acquired using specular X-ray rocking scans to compensate for the missing data in GIWAXS images. For all three tissues, Bragg reflections as partial arcs are seen along both the out-of-plane and the in-plane directions, corresponding to diffraction emanating perpendicular to and along the plane of the cell wall, respectively.

**Fig. 2:**
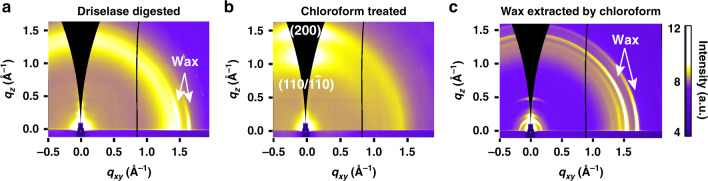
GIWAXS of chemically treated onion epidermal cell wall decouples scattering from cellulose and epicuticular wax crystals. GIWAXS data from **a** Driselase-digested onion epidermal cell wall, **b** chloroform-treated onion epidermal cell wall, and **c** drop-cast wax that was extracted from the onion epidermal cell wall by chloroform.

To probe the origins of the reflections seen in GIWAXS images, we treated onion cell walls with Driselase and chloroform. Driselase is an enzyme that removes all polysaccharides including cellulose, pectins, and hemicelluloses, leaving behind the cuticle. In Fig. 2a, GIWAXS data of Driselase-digested onion cell wall retain the reflections in the in-plane direction seen in unextracted onion walls but no longer show two bright arcs in the out-of-plane direction seen in Fig. 1b. Instead of the arcs in the out-of-plane direction, there is a faint and azimuthally isotropic band. This implies that cellulose crystals are completely digested, and amorphous residues of the digested cell walls contribute to this broad peak. Furthermore, epicuticular wax is known to be crystalline^29,30^, and in-plane features near *q* = 1.5 Å^−1^ and *q* = 1.7 Å^−1^ (Fig. 2a, c) agree with previously reported XRD data from platelet waxes^29^. We thus attribute the in-plane GIWAXS peaks to cuticular waxes.

**Fig. 3:**
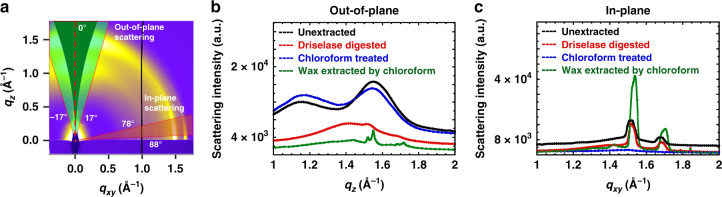
Differences in GIWAXS out-of-plane and in-plane scattering profiles and peak locations suggest multiple diffracting species. **a** Reduced GIWAXS profiles are obtained by azimuthally integrating over a sector from −17° to +17° (area highlighted in green) along the out-of-plane direction and from 78° to 88° (area highlighted in red) along the in-plane direction. **b**, **c** GIWAXS profiles derived from onion epidermal cell walls with different treatments (unextracted: black, Driselase digested: red, chloroform treated: blue, wax extracted by chloroform: green) obtained from sector averages along the **b** out-of-plane direction (sector average from −17° to +17°) and **c** in-plane direction (sector average from 78° to 88°).

Scattering profiles were reduced from 2D images by azimuthally integrating over sectors along the out-of-plane direction (−17° to 17°) and the in-plane direction (78° to 88°), as shown in Fig. 3a. GIWAXS data in the out-of-plane direction from primary cell wall samples (Supplementary Fig. 3a) have peaks around scattering vectors *q* = 1.15 Å^−1^ and *q* = 1.55 Å^−1^ (*q* = 4*π* Sin(*θ*/2)/*λ*, where *θ* is the scattering angle and *λ* is the X-ray wavelength) for onion and Arabidopsis hypocotyls. The peak positions for the out-of-plane reflections of moss leaves are slightly different, at approximately *q* = 1.13 Å^−1^ and *q* = 1.57 Å^−1^. The in-plane reflections from the three different cell walls (Supplementary Fig. 3b) are slightly different from each other. GIWAXS data from onion show two sharp in-plane reflections near *q* = 1.52 Å^−1^ and *q* = 1.68 Å^−1^, Arabidopsis hypocotyls show one sharp in-plane reflection at *q* = 1.52 Å^−1^, while data from moss show two in-plane reflections at *q* = 1.46 Å^−1^ and *q* = 1.54 Å^−1^.

After chloroform treatment, the GIWAXS in-plane features disappear, but out-of-plane features remain intact (Figs. 2b and 3b), as compared to unextracted onion (Fig. 1b). Sharp peaks are not observed from the in-plane scattering profile in Fig. 3c; instead, a weak shoulder is present near *q* = 1.5 Å^−1^. The loss of sharp peaks at *q* = 1.5 Å^−1^ and *q* = 1.7 Å^−1^ is also observed in the azimuthally integrated WAXS data (Supplementary Fig. 4). Altogether, the GIWAXS and WAXS data suggest that chloroform treatment of the cell wall removes crystalline wax.

Wax crystals were also reconstituted by drop casting the chloroform-extracted solution onto silicon wafers. The GIWAXS image (Fig. 2c) from reconstituted wax has similar in-plane features as the unextracted (Fig. 1b) and Driselase-digested (Fig. 2a) onion epidermal cell wall. The in-plane scattering profiles in Fig. 3c indicate that both the peak locations and the shape of the peaks of the reconstituted wax are slightly different than the native wax in unextracted and Driselase-digested epidermis. We speculate that this is a result of re-crystallization of the wax from an organic solvent as opposed to the native state of cuticle-associated wax, given that organic crystal structures are sensitive to the crystallization process and the local environment^40,41^.

SFG spectroscopic studies indicate that onion cellulose is dominated by the Iβ allomorph^42^, such that the (110), ($1\bar 10$), and (200) planes should produce diffraction peaks at *q* = 1.05 Å^−1^, *q* = 1.18 Å^−1^, and *q* = 1.62 Å^−1^, respectively^43^ (see Supplementary Table 1). We attribute the broad peak near *q* = 1.15 Å^−1^ in the out-of-plane GIWAXS profile from onion (Fig. 3b) as combined (110) and ($1\bar 10$) reflections. Such overlap of (110) and ($1\bar 10$) reflections has been observed in bleached softwood^44^, spruce wood^7^, celery collenchyma^12^, sugarcane bagasse^45^, mung bean cell wall^9^, and *A. thaliana* seedlings^46^ with XRD and WAXS. Part of the peak overlap is likely due to small changes in the unit cell beta angle^47^. Peak broadening can arise from many factors, such as limited instrumental resolution, paracrystallinity, and small crystallite size^48–53^; small crystal size has been implicated as the main culprit for diffraction peak broadening of plant cell walls^54^. We attribute the peak near *q* = 1.55 Å^−1^ as the (200) reflection, although it is lower than the value (*q* = 1.62 Å^−1^) for the reported structure of cellulose Iβ^43^. The spacing between (200) planes (*d* = 4.05 Å, *d* = 2*π*/*q*) of the onion epidermal cell wall determined from GIWAXS falls in the range of values reported for mung bean cell walls (*d* = 4.10 Å)^9^ and spruce wood (*d* = 4.00 Å)^7^. This variability observed in *d*-spacing of (200) planes might be due to higher disorder in cellulose crystals of plant cell walls compared to tunicate cellulose, which was used to solve the unit cell dimensions of cellulose Iβ^43^.

We compare our GIWAXS pattern to expected diffraction from cellulose Iβ using GIXSGUI, a software used for visualization and data reduction for grazing incidence X-ray scattering^55^. Bragg reflections simulated by GIXSGUI overlap GIWAXS data from onion cell wall, as shown in Supplementary Fig. 5a. The cell wall appears to contain two populations of crystals, leading to the (200) or (110/$1\bar 10$) plane stacked in the direction normal to the plane of the cell wall. The off-meridional reflections of either population are not observed, which we speculate is a signature of significant in-plane disorder, as discussed below. Comparing GIWAXS profiles with predicted diffraction for a crystal size of 3 nm using MAUD, a Rietveld refinement tool^56^, shows reasonable agreement (Supplementary Fig. 5b). We speculate that the slight shifts in peak positions with respect to tunicate cellulose Iβ are due to the mixture of cellulose Iα and cellulose Iβ found in higher plants^57^ or because cellulose crystals in tunicates are much larger and more ordered than in plants^58^.

### Quantifying degree of preferred crystal orientation through X-ray pole figures

The uniform distribution of intensities along scattering rings in transmission WAXS data (Supplementary Fig. 1) suggests an isotropic orientation of microfibrils along the plane of onion cell walls. Furthermore, GIWAXS patterns (Supplementary Fig. 6) do not change significantly upon rotation in steps of 45 degrees in the *xy*-plane (plane of the cell wall). Thus *q*_*x*_ and *q*_*y*_ are equivalent and cell wall samples are in-plane isotropic on the length scale illuminated by the incident X-ray beam (roughly 10^5^ µm^2^).

Taking advantage of the in-plane isotropy, we can represent the distribution of crystal orientations through *χ*-pole figures, where *χ* is the angle normal to the substrate, by representing the polar angle dependence of a given reflection. *χ*-pole figures from GIWAXS of plant cell walls can depict the population of crystals oriented with respect to the plane of the cell wall; as shown in Fig. 1, however, GIWAXS data are missing information along the out-of-plane direction. Flat 2D detectors cannot intercept the entire Ewald sphere due to the grazing incidence geometry, such that crystallites that are oriented perfectly parallel to the substrate are not sampled in GIWAXS^24^.

**Fig. 4:**
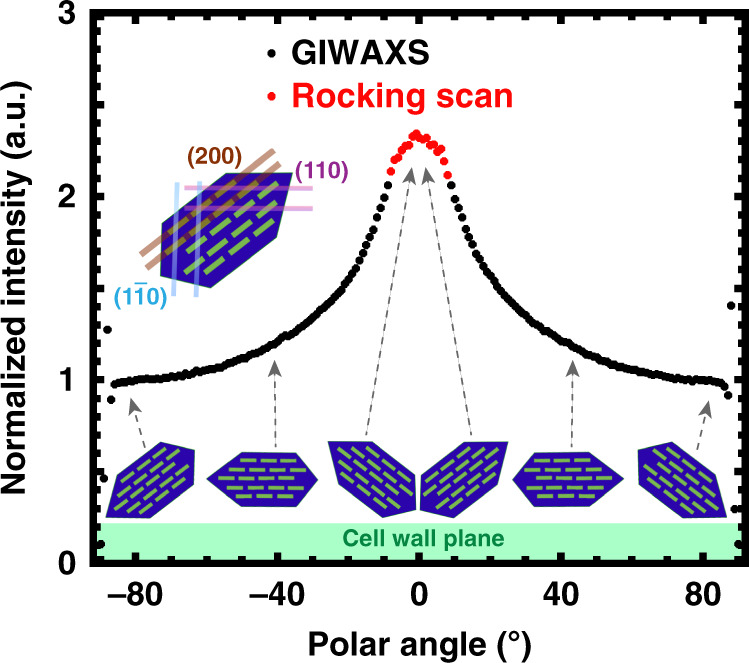
*χ *- pole figure constructed from a combination of GIWAXS and a rocking scan. GIWAXS (black) and rocking scan (red) data are combined to generate a *χ*-pole figure at the cellulose (110)/($1\bar 10$) reflection from the unextracted onion epidermal cell wall. Schematic shows the orientation of cellulose crystallites relative to the cell wall plane as implied by the pole figure from onion epidermis. Inset: cross-section of cellulose microfibril highlighting relevant crystal planes.

We construct *χ*-pole figures through combinations of local specular rocking scans with GIWAXS to sample all crystallite orientations. Rocking the sample within a few degrees of the Bragg reflection angle integrates over crystallites that are not aligned exactly parallel to the substrate^19,20,59^. This approach has been demonstrated for polymeric thin films with a fiber texture, where the crystallite orientation distribution is isotropic in the plane of the substrate^22,24,59–63^. A *χ*-pole figure of (110)/($1\bar 10$) crystal planes for fifth scale unextracted onion epidermis is shown in Fig. 4, where a preferential orientation along the out-of-plane direction is apparent. Integrating the area under the pole figures allows quantification of the degree of preferred orientation, and we find that about 50% of the (110)/($1\bar 10$) planes are oriented between −34° and +34° from the substrate normal. An illustration of the relative orientation of (110)/($1\bar 10$) planes with respect to the cell wall plane as implied by the intensities at specific polar angles is also shown schematically in the bottom of Fig. 4. Similar χ-pole figures for Arabidopsis hypocotyls and moss are shown in Supplementary Fig. 7. A preferred crystal orientation with respect to the plane of the cell wall is clear for all primary cell walls examined in this study.

### Lack of cellulose-preferred orientation in ground cell walls and at the base of growing region in 6-week-old Arabidopsis inflorescence stems

Chloroform-treated onion epidermal cell walls were ground using a vertical vibrator mill to reduce the inherent cellulose-preferred orientation and were used in GIWAXS experiments. The crystallinity index, computed from the Segal method^64^, of intact chloroform-treated onion cell wall is 29%, while that of ground chloroform-treated cell wall is 26%, consistent with previous reported values for onion of 31%^65^. GIWAXS data shown in Supplementary Fig. 8a from ground cell wall reveal a much more isotropic pattern as compared to the onion epidermis seen in Fig. 1b. The break-up of plant tissues into small particles that can be randomly oriented leads to isotropic scattering and significantly diminished signatures of preferred crystal orientation of cellulose, as expected. The diminished signature of a preferred orientation for cellulose is not likely to be due to reduced crystallinity, given the similar values obtained from the Segal method.

We also collected GIWAXS data from segments 1 cm in length from the base of the growing region of inflorescence stems of 6-week-old Arabidopsis plants. These segments are designated as segment #1 to segment #4 as previously reported^66^, where segment #4 is toward the base of the Arabidopsis inflorescence stem. Segment #4 is also where secondary cell wall formation begins. GIWAXS data from segment #4 are shown in Supplementary Fig. 8b. We propose two hypotheses that could lead to isotropic GIWAXS intensities from inflorescence stems. One, the tissue geometry may allow for geometrical averaging of scattering from cellulose. Inflorescence stems resist flattening, unlike onion peels, Arabidopsis hypocotyls, and moss leaves. A cylindrical arrangement of cell walls would lead to isotropic GIWAXS peaks. Another possibility is that microfibrils are twisted or that the net orientation of cellulose crystals in secondary cell wall is less pronounced than in primary cell walls. Indeed, previous work has shown differences in the cellulose content and microfibril arrangement between segment #4, where secondary cell wall starts to form, and segment #1, which consists of primary cell walls^66^.

### Grazing incidence small-angle X-ray scattering (GISAXS) of primary cell walls in onion epidermis and Arabidopsis hypocotyls

GISAXS can complement GIWAXS by providing structural information on the length scale of a few nanometers to hundreds of nanometers^19,21^. GISAXS data from onion epidermal cell wall and Arabidopsis hypocotyls are shown in Supplementary Fig. 9. Although integrated profiles from onion cell walls do not show any apparent features (Supplementary Fig. 9c, d), profiles from Arabidopsis hypocotyls show stronger scattering, a broad feature at about *q* ~ 0.1 Å^−1^ along the out-of-plane direction (Supplementary Fig. 9c), and a weak feature at about *q* ~ 0.15 Å^−1^ along the in-plane direction (Supplementary Fig. 9d). These features correspond to length scales (2*π*/*q* about 4–6 nm) that are consistent with the size of microfibrils in primary cell walls^11,67^. Given that GISAXS peaks arise from periodicities in the sample, we speculate that the 4–6-nm length scale arises when microfibrils pack tightly in bundles. We further hypothesize that the lack of scattering features in the small-angle regime for onion implies that cellulose microfibrils are more bundled or more regularly packed in bundles in Arabidopsis hypocotyls than in onion cell walls.

## Discussion

**Fig. 5:**
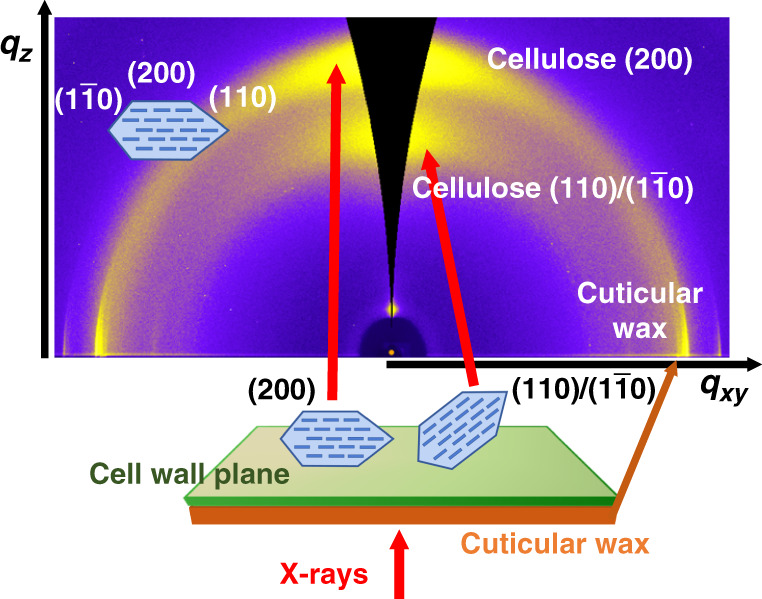
Schematic of crystal orientation of cellulose with respect to the plane of the cell wall. The out-of-plane grazing-incidence scattering from primary cell walls arises from preferentially oriented cellulose crystals, whereas in-plane scattering arises from epicuticular wax crystals. Crystal planes that lead to out-of-plane reflections are denoted in the two populations of cellulose crystals shown. The presence of both (110)/($1\bar 10$) or (200) along the out-of-plane direction in GIWAXS data suggests that two populations of crystallites are present in primary cell walls, each with either (110)/($1\bar 10$) or (200) planes stacked parallel to the plane of the cell wall. Non-crystalline polysaccharides, such as pectin and hemicellulose, are not shown. Inset, top left: cross-section of cellulose crystals with labeled crystal planes.

GIWAXS data after various treatments of the onion epidermal cell wall reveal that in-plane scattering arises from epicuticular wax crystals. We speculate that this in-plane orientation is a consequence of layered wax structures lying on the plane of the cell wall, which have been previously identified through electron and XRD studies^29^. Out-of-plane scattering arises from preferentially oriented cellulose crystals, as shown in the schematic in Fig. 5. These findings suggest that two populations of crystals are oriented such that the (200) or (110) planes are mostly stacked in the direction normal to the cell wall plane. One possibility is that these populations are segregated into separate lamella within the cell wall. The texturing of crystallites allows GIWAXS to decouple scattering from cellulose and epicuticular wax crystals in primary cell walls, enabling their independent study. An alternative approach to independently examine cellulose is through chloroform treatment to remove wax; but Fig. 3b shows that the location of the (110)/($1\bar 10$) and (200) reflections and the shape of GIWAXS out-of-plane scattering profiles of the chloroform-treated samples differ slightly from unextracted samples. The slight differences in peak shape and location could be due to a change in crystalline structure of cellulose introduced by interaction of chloroform with hydrophobic faces of cellulose crystals or because of solvent-based dehydration that could differ from ambient drying of cell walls.

Twisting of microfibrils has been observed through simulations^25,26,68–74^ and experiments^12,18,25^. The twist observed in simulations has been attributed to van der Waals interactions that aid in packing of crystals between layers^75^ or *trans*-glycosidic linkages due to hydrogen bonds^76^. Macroscopic twisting of bacterial microfibrils composed of cellulose has been observed experimentally through direct visualization. Left-handed or right-handed helically twisted bacterial microfibrils were observed during cellulose synthesis in *Acetobacter xylinum* using transmission electron microscopy (TEM)^18,77–79^. Local twisting of microfibrils was also observed through AFM and TEM of *Micrasterias denticulata* cell walls^80^ and through electron microdiffraction of aqueous suspensions of tunicate cellulose nanocrystals^81^. In addition, the cellulose suspension extracted from wood pulp forms a chiral nematic suspension in water^82^. Helical twisting of cellulose microfibrils has also been observed in molecular dynamics simulations of cellulose Iβ^25,68,70,71,73,83^ and Iα^72^ and in density functional theory (DFT) calculations on cellulose Iβ^26,69^. The reported degree of twist is mostly from simulations, with ranges between 0.2 and 9 degrees/nm^70,71,75,83^, and from a few experimental reports, which report values between 3.5 × 10^−3^ and 1 degrees/nm^25,84^. Nevertheless, anisotropic scattering in GIWAXS as seen in Fig. 1 indicates that cellulose microfibrils are not twisted in these three primary cell walls. *χ*-pole figures show that cellulose crystals have a preferred out-of-plane orientation with respect to the cell wall plane, as shown in Fig. 4. Twisted cellulose microfibrils would create a net isotropic orientation of cellulose crystals, yielding isotropic rings in scattering profiles and pole figures that lack a preferred orientation. Lack of twisting of cellulose microfibrils is consistent with an AFM-based study of cellulose microfibrils in onion epidermis^13^.

The loss of texturing observed after grinding of cell walls (Supplementary Fig. 8a) demonstrates that the apparent texture is not an artifact of the scattering instruments. This is further support by the GIWAXS data from segment #4 (base of growing region where secondary cell wall begins to form) of inflorescence stems of 6-week-old Arabidopsis plants that show isotropic scattering (Supplementary Fig. 8b), which could be a result of the tissue geometry (random orientation of cell walls), a random orientation of cellulose crystals, or twisting of cellulose microfibrils.

We hypothesize that the physiological environment in which cellulose microfibrils are deposited is important for the preferred crystal orientation. In plant primary cell walls, cellulose microfibrils are synthesized by the cellulose synthase complex and are then deposited into an amorphous polysaccharide matrix^85–87^. A preferred orientation of cellulose crystals relative to the plane of the cell wall supports the hypothesis that cellulose synthase complexes do not rotate within the plasma membrane^88^. Furthermore, a previous TEM study has shown that carboxymethyl cellulose (CMC), a negatively charged water-soluble cellulose derivative, reduces twisting of microfibrils secreted by *Acetobacter xylinum*^78^. Low degrees of substitution in CMC enable it to closely associate with native cellulose, thus affecting cellulose assembly. Some of the hemicelluloses in plant cell walls are structurally similar to CMC and could have a similar effect on cellulose microfibril twisting in plant cell walls, although DFT calculations suggest that xylan does not interact with cellulose strongly enough to affect conformations^89^. Nevertheless, recent AFM studies of onion epidermis^13,14,90,91^, celery parenchyma walls^92^, celery collenchyma^12^, and maize primary cell walls^93^ show no evidence of cellulose microfibril twisting. Correlating experimental δ^13^C NMR chemical shifts with chemical shifts for twisted cellulose microfibrils predicted using DFT suggests that twisting is unlikely or very minor (at most 0.2 degrees/nm)^26^.

The out-of-plane GIWAXS data (Fig. 3) suggest that two populations of crystallites are present in primary cell walls, each with either (110)/($1\bar 10$) or (200) planes stacked in the direction normal to the plane of the cell wall. Thus, we expect a complementary reflection to be present away from the out-of-plane direction, at an angle that is consistent with the unit cell structure (for example, see Supplementary Fig. 5 for when (200) planes are stacked out of plane). This is not observed in our GIWAXS data (Fig. 1). We hypothesize that chain packing within microfibrils, and degree of order, is strongest in the out-of-plane direction, such that more disorder along the plane of the cell wall leads to weaker diffraction that is not apparent in our data. Comparing azimuthally integrated WAXS profiles and sector integrated GIWAXS profiles (out-of-plane, in-plane, Supplementary Fig. 4) shows that peak positions of (200) reflections differ (WAXS at *q* = 1.52 Å^−1^, GIWAXS out-of-plane at *q* = 1.55 Å^−1^, and GIWAXS in-plane at *q* = 1.5 Å^−1^). Because transmission WAXS probes planes stacked in directions along the cell wall plane, whereas out-of-plane GIWAXS probes the direction normal to the cell wall plane, this difference in peak position indicates that (200) planes are more tightly packed within microfibrils in the direction normal to the cell wall plane (*d* = 4.2 Å) than along the plane of the cell wall (*d* = 4.05 Å). We speculate that this is a signature of less densely packed cellulose in-plane when compared to the out-of-plane direction.

In cellulose crystals, the surfaces of (200) planes are hydrophobic, whereas surfaces of (110)/($1\bar 10$) planes are hydrophilic^12^. Hydrophobic–hydrophobic and hydrophilic–hydrophilic interactions are likely to be important for inter-crystallite interactions and cell wall mechanics. It has been proposed that wall extensibility is controlled by sites of close contact between cellulose microfibrils, potentially mediated by trace amounts of xyloglucans. These regions are called “biomechanical hot-spots”^94,95^. Because xyloglucan is hypothesized to bind preferentially to hydrophobic surfaces of cellulose crystallites^74,96^, it is possible that the preferred orientation of crystallites could promote formation of the proposed biomechanical hot-spots through hydrophobic–hydrophobic interactions. A preferred orientation of cellulose crystals could also lead to formation of large hydrophobic or hydrophilic surfaces that could be important for mediating interactions with matrix polysaccharides and for promoting a network capable of propagating stress throughout primary cell walls.

Even though the out-of-plane and in-plane scattering from primary cell walls of onion epidermis, Arabidopsis hypocotyls, and moss look similar, there are subtle differences in the Bragg reflections. This suggests structural variations in primary cell walls depending on the source of tissue. The peak positions for the out-of-plane reflections of moss leaves are slightly different from those of the onion epidermal cell wall and Arabidopsis hypocotyls. Because cellulose in land plants is a mixture of cellulose Iα and cellulose Iβ^57,97^, the difference in peak positions could be a reflection of a change in composition of cellulose Iα and cellulose Iβ depending on the source. Bryophytes (moss) and angiosperms (onions and Arabidopsis) are also reported to have subtle differences in cell wall composition^98,99^, which could impact interactions or packing of glucan chains. The (110/$1\bar 10$) planes have broader reflections in moss when compared to onion epidermis and Arabidopsis hypocotyls, suggesting less crystalline cellulose, the presence of more crystal defects, or perturbations to the unit cell beta angle that affect the overlap between the 110 and $1\bar 10$ reflections^47^. The in-plane reflections seen in the three different samples are also slightly different from each other, indicating a difference in crystalline structure of epicuticular wax crystals in the three different species.

The canonical description of cell wall assembly assumes that microfibrils are deposited in random orientations or in a way that allows microfibrils to twist. GIWAXS of plant primary cell walls shows that this description is likely not correct. Instead, we report a new type of cellulose organization, where microfibrils have some coherence in terms of their relative crystal orientation. Our findings, seen so far in primary cell walls from onion epidermis, Arabidopsis hypocotyls, and moss, imply that a general mechanism of cell wall biosynthesis somehow leads to this cellulose crystal texturing. Thus, not only does measuring the orientation of cellulose crystallites relative to the plane of the cell wall reveal a previously unreported aspect of cell wall structure but also implies an aspect of cell wall assembly not previously considered.

## Methods

### Chemical treatments of samples

*Onion epidermal cell wall*: Abaxial fifth scale onion epidermis was peeled from white onion bulbs (*Allium cepa*, cv. Cometa) purchased from a local grocery store. Fifth scale epidermis peel refers to the peel from the onion scale that comes fifth when fleshy scales are numbered consecutively (1,2, 3,…,*n*) from the outside with the first scale as the outermost fleshy scale after removing the dried layers^90^. All peels were washed in 0.1% Tween-20 in 20 mM HEPES buffer (pH 6.8) for 1 h^90^. Driselase powder (a fungal enzyme cocktail; Sigma; Cat # D9515) was dissolved in 20 mM sodium acetate buffer (pH 5.5) for 30 min to make a final concentration of 10 mg/mL. The Driselase solutions were centrifuged at 2500 × *g* for 5 min, and then the supernatant was passed through a 0.2-µm filter to remove any insoluble particles. To examine the cuticle, peels were treated with 10 mg/mL Driselase solution at 37 °C in 20 mM Tris (pH 8.5) for 6 days with gentle shaking at 50 rpm. To extract epicuticular wax from the epidermis, peels were gently stirred in 2 mL of chloroform overnight at room temperature. The epidermis was subsequently collected using tweezers. The chloroform solution was filtered into a glass vial with a 0.45-μm polyvinylidene difluoride filter and then the chloroform was evaporated. Afterwards, the extracted wax was redissolved in 100 μL of chloroform and drop cast on a silicon substrate. The unextracted, Driselase-digested, and chloroform-treated epidermises were then washed with deionized (DI) water six times.

*Arabidopsis hypocotyls*: Hypocotyls of 6-day-old dark grown *A. thaliana* were washed in 0.1% Tween-20 in 20 mM HEPES buffer (pH 6.8) for 1 h with shaking at 50 rpm and then washed with DI water.

*Moss*: Ten moss (*Physcomitrella patens)* leaves (phyllids) were extracted in 1% sodium dodecyl sulfate (SDS) for 24 h and rinsed three times, 10 min each, with DI water.

*Ground onion cell wall*: Abaxial fifth scale onion epidermis peels from white onion bulbs were extracted with chloroform as described above. Thirty onion peels were then finely ground using a vertical vibration mill (Sweco Vibro-Energy Grinding Mill, Model No. GM005) with yttrium-stabilized zirconia balls (diameter: 5 mm) in DI water as a liquid media at 1140 rpm for 72 h. Some solvent was evaporated from the solution containing ground cell wall to form a slurry.

*Arabidopsis inflorescence stems*: *A. thaliana* stem segments were prepared as previously reported^66^. Arabidopsis stem segment 4 was chosen for this experiment. Five Arabidopsis stems were washed in 2% SDS for 12 h, flattened between slides with a 700-g weight, washed in SDS for three additional cycles (48 h total), and then equilibrated in water. The stems were extracted with chloroform under stirring for 24 h to remove epicuticular wax.

### GIWAXS and GISAXS sample preparation

Silicon substrates for GIWAXS and GISAXS experiments were cleaned by sonication in acetone, iso-propanol, and DI water sequentially. For unextracted, Driselase-digested, and chloroform-treated onion GIWAXS samples, a single peel was mounted on a silicon substrate in a hydrated state so that the samples adhered better to the substrate as they air dried. A single peel of unextracted onion cell wall in a hydrated state was mounted on a cleaned silicon substrate for GISAXS measurements as well. Chloroform-extracted epicuticular wax was drop-cast on a silicon substrate for GIWAXS measurements.

For GIWAXS and GISAXS of Arabidopsis, 30 hydrated hypocotyls, each approximately 15 mm long, were mounted flat side by side on a silicon substrate and then air dried. Once dry, hypocotyls are macroscopically flat. For each moss sample, 10 hydrated moss leaves were mounted flat and air dried on a silicon substrate so as to resemble a thin film. The GIWAXS sample of ground onion cell wall was made by drop-casting a slurry of the ground onion cell wall on a cleaned silicon substrate.

Flattened, chemically treated Arabidopsis inflorescence stem segments were mounted on cleaned silicon substrates and air dried. The stems were adhered to the substrate using carbon tape at the edges of the stem segments as they partially dried. For GIWAXS measurements, the inflorescence stem samples were mounted along the beam such that the tape was not in the path of the X-ray beam.

### Wide-angle X-ray scattering

For transmission WAXS experiments, 10 unextracted and 7 chloroform-treated onion epidermal peels, as prepared for GIWAXS experiments, were stacked together on washers with the longitudinal direction of the epidermal cells aligned to enhance scattering intensity.

WAXS experiments were performed at beamline 7.3.3 of the Advanced Light Source (ALS) at Lawrence Berkeley National Laboratory. Samples were loaded in a helium chamber to minimize background scattering. Data were collected using 10 keV X-rays and a Pilatus 2M detector. A sample-to-detector distance of 27.5 cm was used to cover a *q* range of 0.08–3.38 Å^−1^. 2D images were azimuthally averaged to obtain the reported one-dimensional (1D) scattering profiles using the Nika package^100^. Three independent samples were measured for each condition to ensure repeatability.

### GIWAXS and analysis

GIWAXS experiments were carried out at beamline 7.3.3^101^ of the ALS at Lawrence Berkeley National Laboratory and beamline 11-3 of Stanford Synchrotron Radiation Lightsource (SSRL) at SLAC National Accelerator Laboratory. Samples mounted on silicon substrates were examined in a helium environment to minimize background scattering. At ALS, data were collected using 10 keV X-rays and a Pilatus detector with an incident angle of 0.15°. At SSRL, data were collected using 12.7 keV X-rays and a Raxyonics 225 detector with an incident angle of 0.12°. For analysis, GIWAXS 2D images were corrected for the curvature of the Ewald sphere using Xi-cam^102^ for data collected at ALS and using WxDiff^103^ for data collected at SSRL. The out-of-plane scattering profile was obtained by integrating over polar angles (*χ*) from −17° to +17° (where 0° is along the vertical direction), and the in-plane scattering profile was obtained from integrated data with polar angles between +78° and +88°. Three independent samples were measured for each condition to ensure repeatability.

### GISAXS and analysis

GISAXS experiments were carried out at beamline 7.3.3^101^ of the ALS at Lawrence Berkeley National Laboratory. Samples mounted on silicon substrates were examined in vacuum to minimize background scattering. At ALS, data were collected using 10 keV X-rays and a Pilatus detector with an incident angle of 0.15°. For analysis, GISAXS 2D images were reduced to 1D data using Xi-cam^102^. 1D GISAXS scattering profiles along out-of-plane and in-plane directions are obtained from a vertical line cut (width, ∆*q*_*y*_ = 0.005 Å^−1^) centered at *q*_*y*_ ~ 0.012 Å^−1^ and a horizontal line cut (width ∆*q*_*z*_ = 0.005 Å^−1^) at *q*_*z*_ = 0.03 Å^−1^, respectively.

### Rocking scans

Rocking scans were carried out at Experimental Station 11-3 of SSRL at SLAC National Accelerator Laboratory. Data were collected using 12.7 keV X-rays and a Raxyonics 225 detector. For pole figures of the (110)/($1\bar 10$) reflection, samples were rocked at 4.4° ≤ *θ* ≤ 5.9° corresponding to 0.99 ≤ *q* ≤ 1.31 Å^−1^. Three independent samples were measured for each condition to ensure repeatability.

### Pole figures

A pole figure is a representation of the orientation distribution of crystallographic planes in a sample, which illustrates the texture of a material. Partial pole figures with polar angles (*χ*) between −90° to −7.5° and 7.5° to 90° were obtained by azimuthally integrating the (110)/($1\bar 10$) reflection over *q* = 1.15 ± 0.16 Å^−1^ from corrected GIWAXS images. Data for pole figures near the specular direction were obtained from rocking scans. A sector integration was performed for the (110)/($1\bar 10$) reflection at *q* = 1.15 ± 0.16 Å^−1^ within −30° < *χ* < 30° of the 2D rocking scan images. To construct the complete pole figure, a scaling factor was used to scale the background-corrected GIWAXS data to match rocking scan data at *χ* = ±7.5°.

### Background correction

Background correction for *χ*-pole figures was done by subtracting a local background from the azimuthal integration of the cellulose (110)/($1\bar 10$) reflection in both GIWAXS and rocking scan data. An azimuthal integration along polar angle (*χ*) of a region outside the (110)/($1\bar 10$) reflection was selected as the local background. The sector with *q*-range from 0.5 to 0.6 Å^−1^ was selected as local background for GIWAXS data for onion. The sector with *q*-range of 0.6–0.7 Å^−1^ was selected for Arabidopsis and moss. The intensity of the local background at each polar angle was subtracted from the intensities of azimuthal cuts over the (110)/($1\bar 10$) reflection at the corresponding polar angle. Similarly, the regions selected as backgrounds for rocking scans have a *q*-range of 2–2.2 Å^−1^ for onion samples, 1.8–1.9 Å^−1^ for Arabidopsis samples, and 0.7–0.8 Å^−1^ for moss samples. The integrated intensities of these regions were subtracted from the intensities of the azimuthal cuts over the (110)/($1\bar 10$) reflection in rocking scan data at the corresponding polar angle.

### Indexing of GIWAXS patterns

GIWAXS 2D and 1D patterns were indexed by comparison to diffraction patterns for cellulose Iβ simulated in GIXSGUI, a software used for visualization and data reduction for grazing incidence X-ray scattering^55^, and MAUD, a Rietveld refinement tool^56^, respectively. Previously reported unit cell parameters for cellulose Iβ were used for simulating diffraction patterns^43^. The 2D GIWAXS diffraction pattern generated in GIXSGUI was overlaid on GIWAXS 2D data of onion cell wall for comparison. In addition, the 1D powder diffraction pattern generated in MAUD was overlaid on GIWAXS 1D data for onion cell wall for comparison. The 1D diffraction data generated in MAUD was for crystal size 30 Å and the pattern was also refined for background to match experimentally obtained GIWAXS 1D data.

### Reporting summary

Further information on research design is available in the Nature Research Reporting Summary linked to this article.

## Supplementary information

Supplementary Information

Reporting Summary

## Data Availability

The data that support the findings of this study can be found in Supplementary Information and are available on Penn State ScholarSphere at https://scholarsphere.psu.edu/collections/bnz805z87t.
