# Supplementary material for: Preferred crystallographic orientation of cellulose in plant primary cell walls
Source: Nat Commun. 2020 Sep 18;11:4720. doi: 10.1038/s41467-020-18449-x (PMC7501228; doi:10.1038/s41467-020-18449-x)
Supplement: Supplementary file 1 — Supplementary Information [file 41467_2020_18449_MOESM1_ESM.pdf]

Supplementary Information for

Preferred crystallographic orientation of cellulose in plant primary cell walls

Dan Ye[1†], Sintu Rongpipi[1†], Sarah N. Kiemle[2‡], William J. Barnes[2], Arielle M. Chaves[3], Chenhui Zhu[4], Victoria A. Norman[4], Alexander Liebman-Peláez[4], Alexander Hexemer[4], Michael F. Toney[5], Alison W. Roberts[3], Charles T. Anderson[2], Daniel J. Cosgrove[2], Esther W. Gomez[1,6*], Enrique D. Gomez[1,7*]

[†]authors contributed equally

[1]Department of Chemical Engineering, The Pennsylvania State University, University Park, PA 16802, United States

[2]Department of Biology, The Pennsylvania State University, University Park, PA 16802, United States

[3]Department of Biological Sciences, The University of Rhode Island, Kingston, RI 02881, United States

[4]Advanced Light Source, Lawrence Berkeley National Laboratory, 1 Cyclotron Road, Berkeley, CA 94720, United States

[5]Stanford Synchrotron Radiation Lightsource, SLAC National Accelerator Laboratory, Menlo Park, California 94025, United States

[6]Department of Biomedical Engineering, The Pennsylvania State University, University Park, PA 16802, United States

[7]Department of Materials Science and Engineering and Materials Research Institute, The Pennsylvania State University, University Park, PA 16802, United States

[‡]Kiemle current address: Mount Holyoke College, 123 Clapp Laboratory, 50 College Street, South Hadley, MA 01075 USA

*Corresponding author email: ewg10@psu.edu, edg12@psu.edu

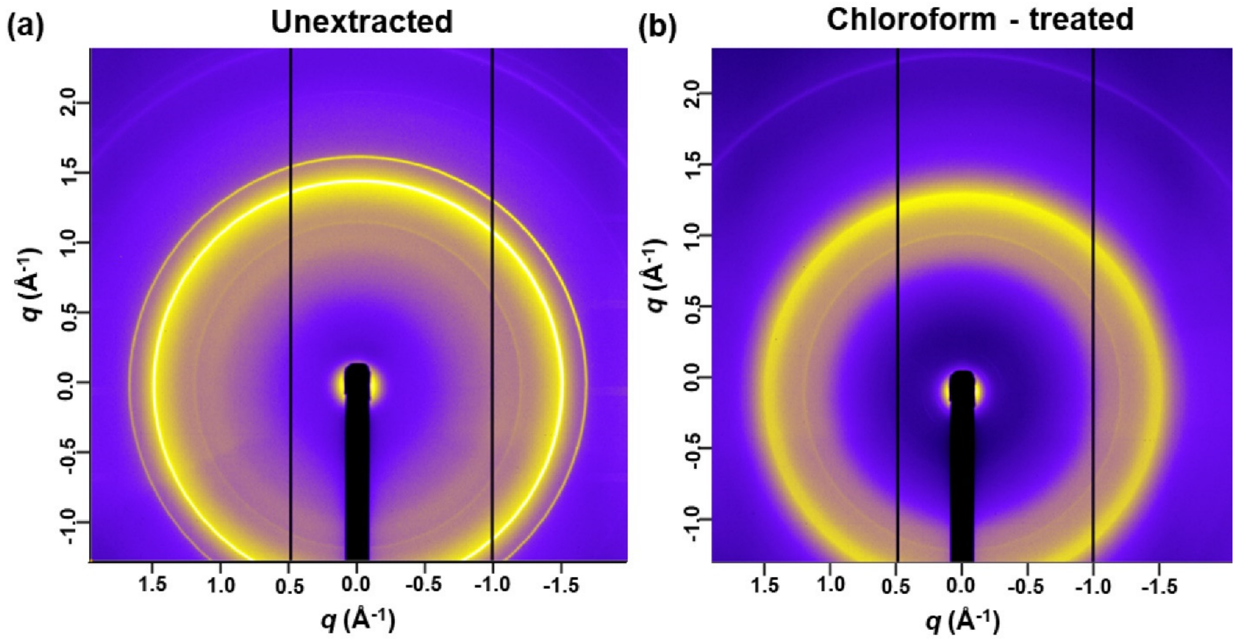

Supplementary Figure 1. Transmission WAXS 2D data from onion epidermis cell wall reveal isotropic scattering. 2D WAXS images of (a) unextracted and (b) chloroform-treated onion epidermal cell walls show diffraction intensities that are uniform with azimuthal angle. Chloroform treatment of onion cell walls leads to the disappearance of the bright rings seen in WAXS data for unextracted onion cell wall. Because chloroform treatment removes epicuticular wax, the data suggest that the bright sharp rings seen in the WAXS data of unextracted onion cell wall are from epicuticular wax crystals. Broader diffraction rings in WAXS data of chloroform-treated onion cell wall are from cellulose crystals.

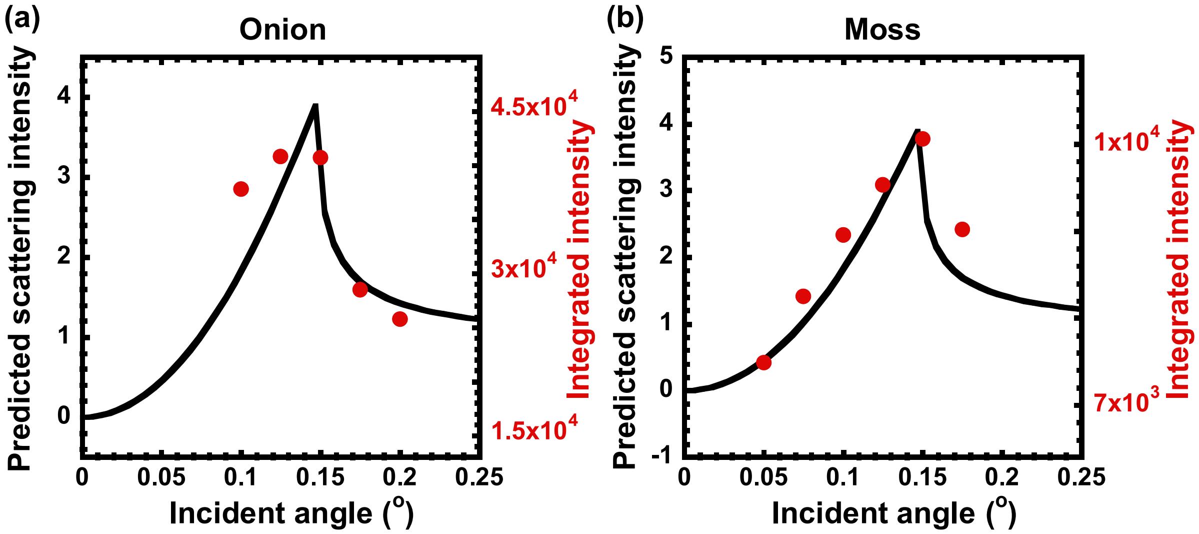

Supplementary Figure 2. A maximum in GIWAXS intensity is observed at the critical angle for total external reflection. Predicted scattered intensity[1] and total integrated GIWAXS intensity versus incident angle for (a) onion epidermis and (b) moss leaves at 10 keV X-rays.

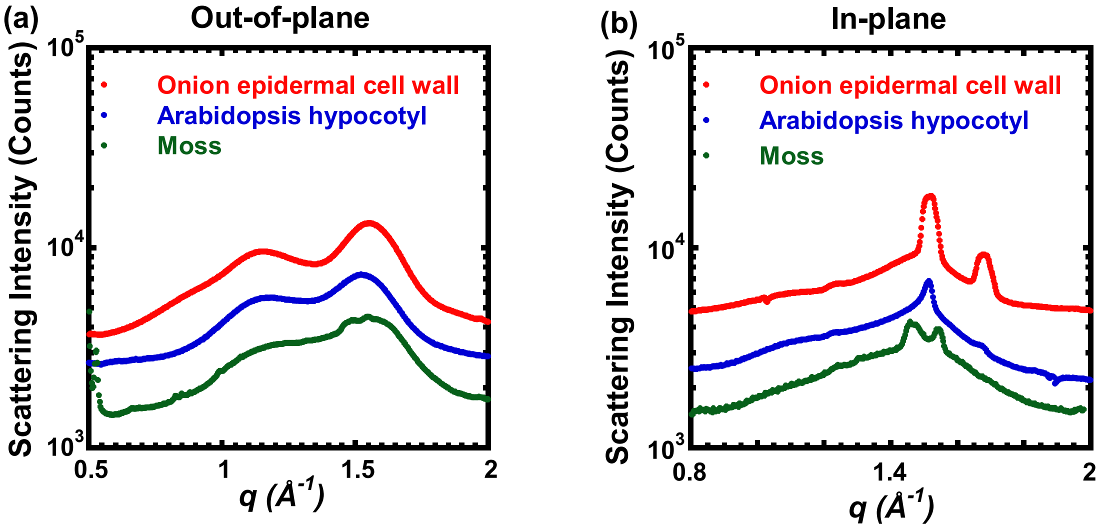

Supplementary Figure 3. GIWAXS out-of-plane and in-plane scattering profiles from primary cell walls in onion, Arabidopsis hypocotyl, and moss are similar. Comparison of sector integrated scattering along (a) out-of-plane direction (sector average from -17° to +17°) and (b) in-plane direction (sector average from 78° to 88°). No intensity offsets were applied.

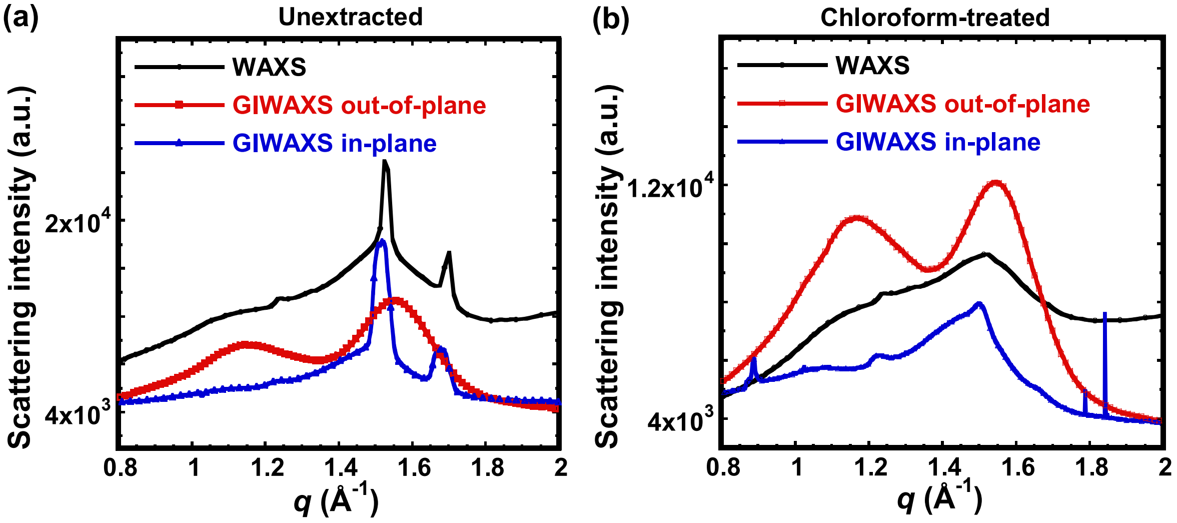

Supplementary Figure 4. Loss of sharp reflections in transmission WAXS and GIWAXS in-plane profiles from chloroform-treated onion cell wall suggests that chloroform treatment of the cell wall removes crystalline wax in the cuticle. Comparison of the azimuthally integrated WAXS profiles and sector integrated (out-of-plane, in-plane) GIWAXS profiles of (a) unextracted and (b) chloroform-treated onion epidermal cell wall.

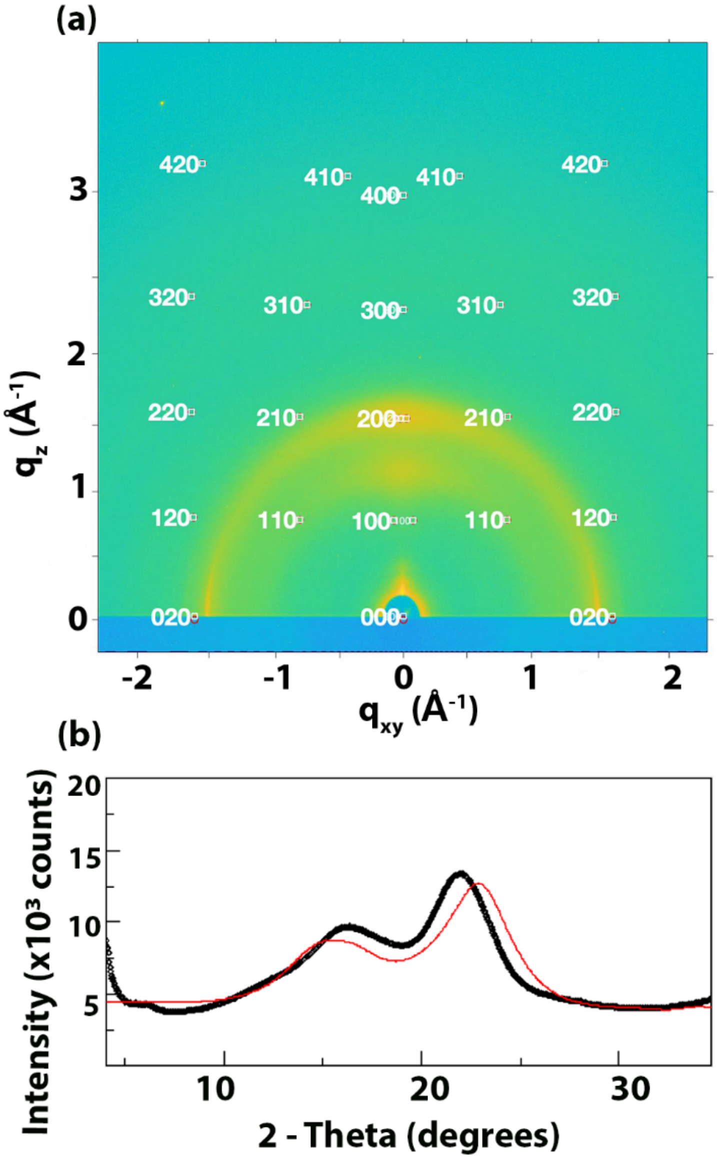

Supplementary Figure 5. Indexing GIWAXS 2D and 1D diffraction patterns confirms GIWAXS out-of-plane scattering is from cellulose. (a) Bragg reflections simulated in GIXSGUI are overlaid on a GIWAXS 2D image of onion cell wall. The GIWAXS image is not corrected for the grazing-incidence geometry (*i.e.*, "missing wedge" seen in Figure 1 of the main text). (b) XRD pattern (red line) of cellulose Iβ (crystal size 30 Å) simulated in MAUD Rietveld program compared to the out-of-plane 1D scattering profile (black line) obtained from GIWAXS of onion cell wall. The simulated diffraction pattern in both cases is obtained using unit cell parameters reported by Nishiyama et al., *J. Am. Chem. Soc.* (2002), 124, 31, 9074-9082.

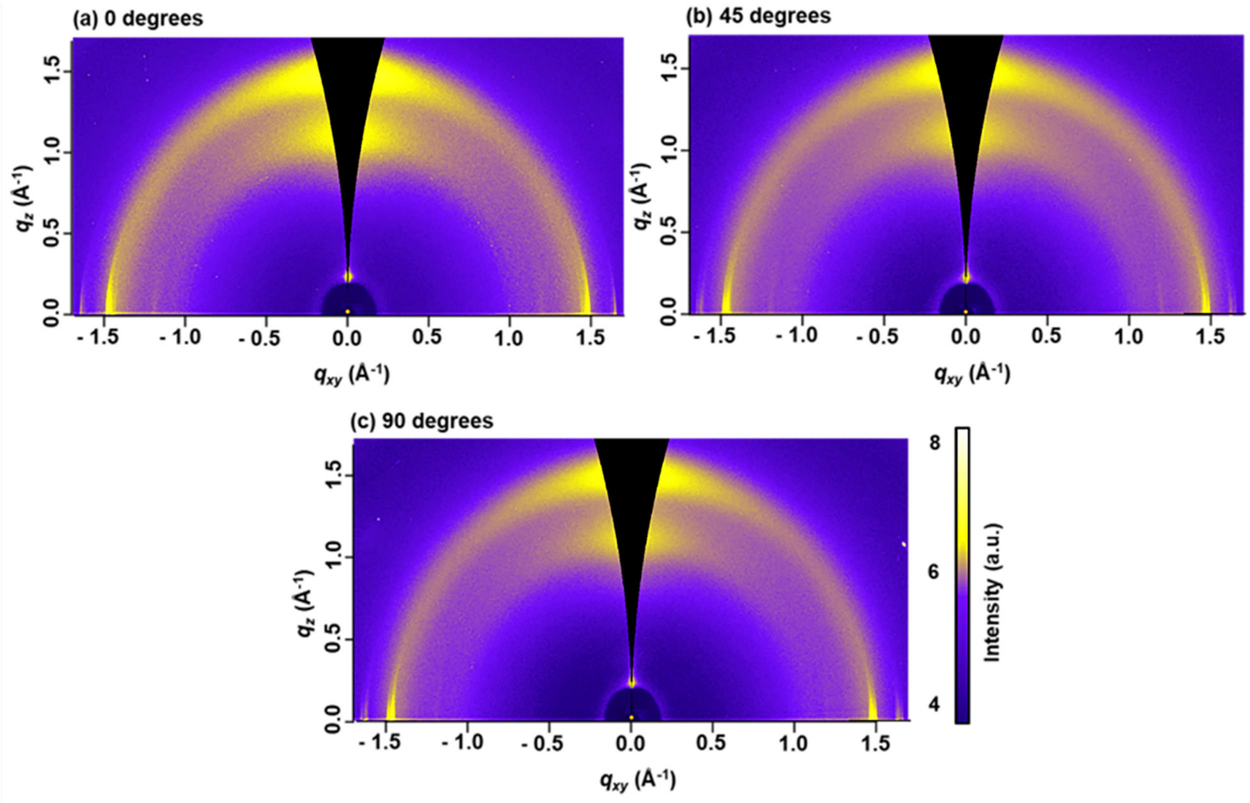

Supplementary Figure 6. GIWAXS 2D data do not change upon in-plane rotation of an onion cell wall sample. 2D GIWAXS images of onion epidermis cell walls after rotating (a) 0 degrees, (b) 45 degrees, and (c) 90 degrees. Data are nearly identical demonstrating the in-plane isotropic alignment of cellulose microfibrils.

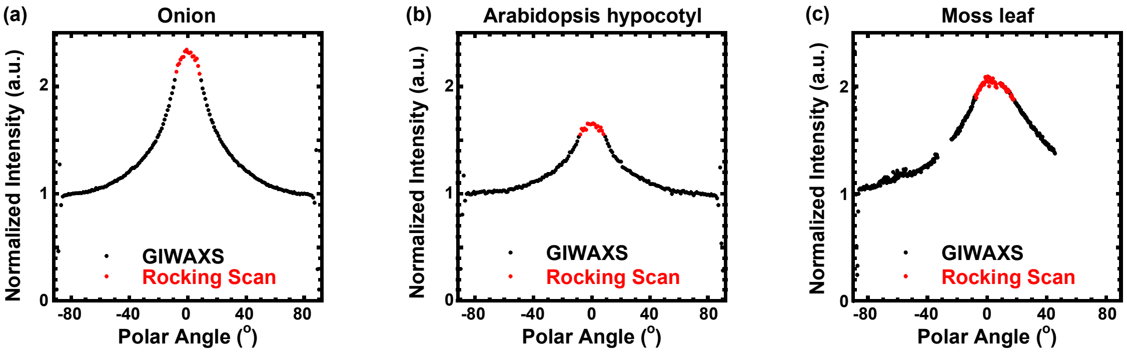

Supplementary Figure 7. χ-pole figures constructed from a combination of GIWAXS and rocking scan confirm the presence of cellulose texture in primary cell walls of onion epidermis, Arabidopsis hypocotyl and moss. Pole figure at cellulose (110)/(1$\bar{1}$0) reflections from (a) unextracted onion epidermis, (b) unextracted Arabidopsis hypocotyl and (c) unextracted moss leaves.

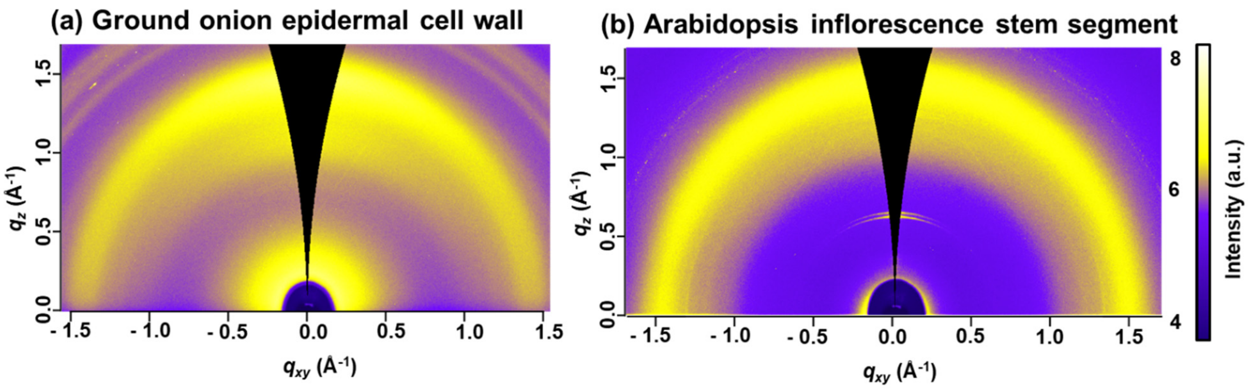

Supplementary Figure 8. GIWAXS 2D data from ground onion cell wall and secondary cell walls of Arabidopsis inflorescence stems reveal a nearly isotropic azimuthal spread of cellulose reflections, suggesting a loss or lack of cellulose texture. 2D GIWAXS images of (a) vertical vibrator milled onion epidermal cell wall and (b) 6-week-old Arabidopsis inflorescence stem. The ground onion cell wall is nearly isotropic due to break up of the cell wall tissue into randomly-oriented particulates. Diffraction from Arabidopsis inflorescence stem is nearly isotropic due to a cylindrical arrangement of the cell wall within the tissue, a loss of texturing due to a random arrangement of microfibrils, or twisting of cellulose microfibrils in secondary cell walls.

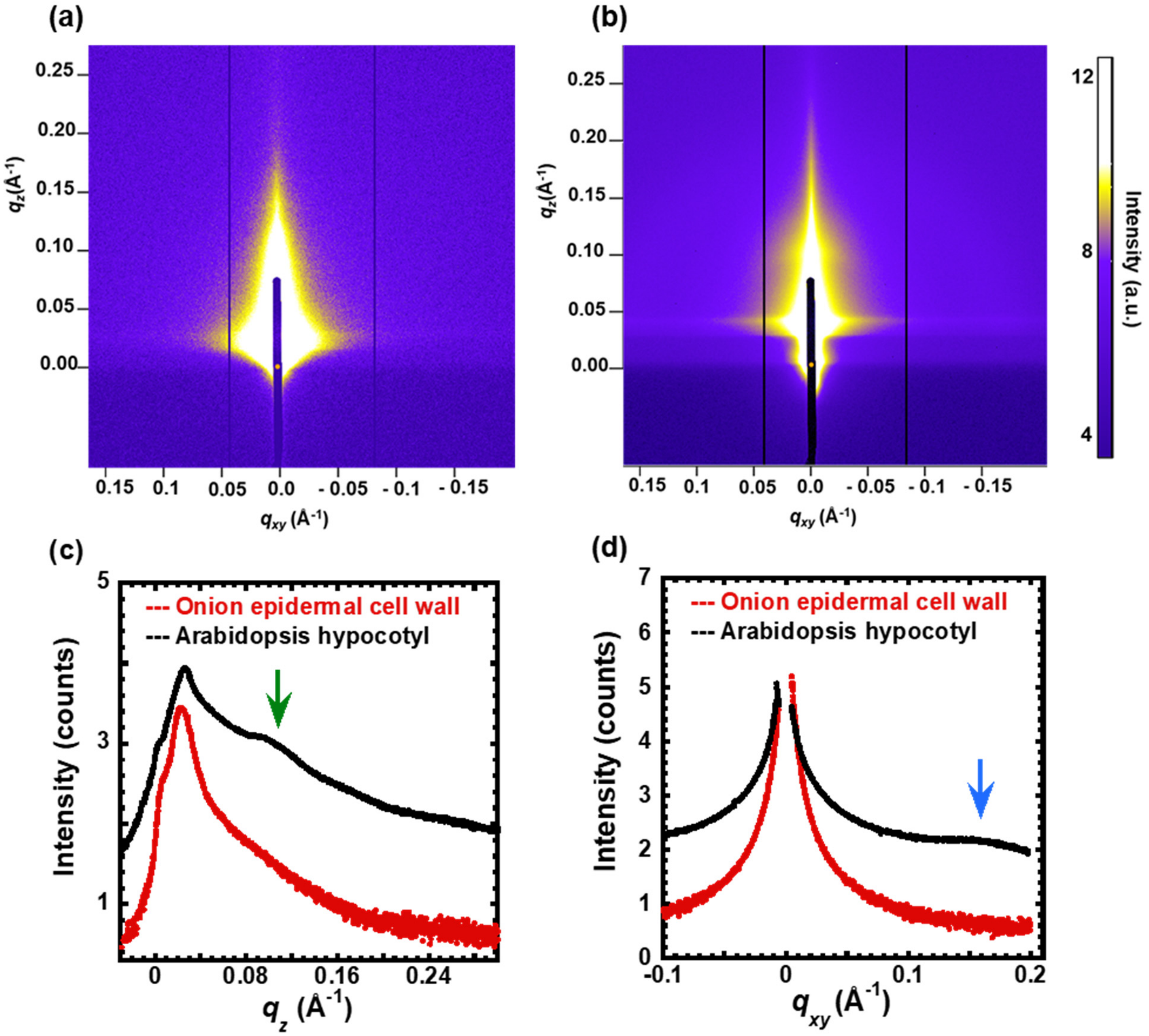

Supplementary Figure 9: GISAXS of primary cell walls of Arabidopsis hypocotyl reveal a scattering feature at the length scale consistent with the size of cellulose microfibrils or microfibril bundles. GISAXS 2D image of (a) unextracted onion cell wall and (b) Arabidopsis hypocotyl. Comparison of 1D GISAXS profile of unextracted onion cell wall (red) and Arabidopsis hypocotyl (black) along (c) out-of-plane direction obtained from a vertical line cut (width Δq_y = 0.005 Å^{-1}) centered at q_y = 0.012 Å^{-1}, and (d) in-plane direction obtained from a horizontal line cut (width Δq_z = 0.005 Å^{-1}) centered at q_z = 0.03 Å^{-1}. GISAXS of Arabidopsis hypocotyl shows a feature at $q_z \sim 0.1$ Å^{-1} (green arrow, corresponding to a length scale of ~6.3 nm) along the out-of-plane direction and at $q_y \sim 0.15$ Å^{-1} (blue arrow, corresponding to a length scale of ~ 4.2 nm) along the in-plane direction. GISAXS of onion cell wall does not reveal any clear scattering peaks.

Supplementary Table 1: Comparison of diffraction peak positions in out-of-plane GIWAXS from onion cell wall with predicted unit cell parameters of cellulose Iβ and peak positions reported from transmission wide angle X-ray scattering of primary cell walls in mung bean and celery.

GIWAXS of onion cell wall	**planes**	**(110)/(1$\bar{1}$0)**		**(200)**	**(004)**
	q (Å^{-1})	1.15		1.55	2.41
Cellulose Iβ (theoretically calculated from reported unit cell parameters[2])	**planes**	**(1$\bar{1}$0)**	**(110)**	**(200)**	**(004)**
	d (Å)	5.96	5.32	3.87	2.60
	q (Å^{-1})	1.05	1.18	1.62	2.42
WAXS of mung bean[3]	**planes**	**(110)/(1$\bar{1}$0)**		**(200)**	
	q (Å^{-1})	1.11		1.54	
WAXS of celery cell wall[4]	**planes**	**(110)/(1$\bar{1}$0)**		**(200)**	
	q (Å^{-1})	~1.1		1.55	

References

1. Renaud G, Lazzari R, Leroy F. Probing surface and interface morphology with Grazing Incidence Small Angle X-Ray Scattering. *Surface Science Reports* **64**, 255-380 (2009).

2. Nishiyama Y, Langan P, Chanzy H. Crystal Structure and Hydrogen-Bonding System in Cellulose Iβ from Synchrotron X-ray and Neutron Fiber Diffraction. *J Am Chem Soc* **124**, 9074-9082 (2002).

3. Newman RH, Hill SJ, Harris PJ. Wide-Angle X-Ray Scattering and Solid-State Nuclear Magnetic Resonance Data Combined to Test Models for Cellulose Microfibrils in Mung Bean Cell Walls. *Plant Physiology* **163**, 1558-1567 (2013).

4. Thomas LH*, et al.* Structure of Cellulose Microfibrils in Primary Cell Walls from Collenchyma. *Plant Physiology* **161**, 465-476 (2012).
